# Performance and usability evaluation of a mobile health data capture application in clinical cancer trials follow-up

**DOI:** 10.1016/j.tipsro.2022.10.005

**Published:** 2022-11-01

**Authors:** John M.J. Paulissen, Catharina M.L. Zegers, Iverna R. Nijsten, Pascalle H.C.M. Reiters, Ruud M. Houben, Daniëlle B.P. Eekers, Erik Roelofs

**Affiliations:** Department of Radiation Oncology (Maastro), Maastricht University Medical Center+, GROW-School for Oncology and Reproduction, Maastricht, the Netherlands

**Keywords:** Electronic data capture, Mobile application, mHealth, Clinical cancer trials, High quality data collection

## Abstract

Mobile health data capture applications (mHDA’s) may improve communication between healthcare providers and patients. However, there is limited literature about the use of mHDA’s facilitating clinical trials. In this study, the effectiveness of an application, supporting follow-up visits of cancer trial participants was investigated. Twenty participants were provided with an e-questionnaire via the mHDA. Participants rated the usability of the application as high performing (mean Systems Usability Scale 87 points). The research team rated the mHDA as highly applicable and efficient in preparing visits. Anamnesis, physical examination and agreement on further policy were performed within an average of 31 min.

## Highlights

• A mobile health data capture application can improve quality of data collection in clinical cancer trials.
• The use of a mobile health data capture application optimizes the efficiency and effectivity of a trial participant’s visit to a clinic.
• A mobile health data capture application relieves participants from instant recall on possible health changes during visits.
• The use of a mobile health data capture application helps a healthcare provider in preparing a participant’s visit.

## Introduction

Clinical trials are essential for evidence-based and safe introduction of new therapies [1], [2]. To provide essential evidence, a robust and highly qualitative data capture in the course of clinical trials is fundamental [1], [3]. Participation and adherence to clinical trial procedures can be demanding, especially for cancer patients because of the recurring confrontation with a potential life-threatening disease [4], [5], [6]. This can cause a significant burden with a typical dropout rate of 25 % for clinical cancer trials (CCTs) [7], [8], [9].

In CCTs, data collection on the effects of treatment may start before treatment and may continue for many months to years in order to obtain a complete data registration. Participants commit themselves to this continuing investment when consenting to the trial and regular visits to a healthcare facility can be necessary for data collection. During these visits, part of the data is collected by questioning participants about their health status over the past period. Participants have to recall this information instantly, including changes in medication and new or different treatments. An incomplete recall of information may lead to an incomplete registration. Trial specific physical examinations can be part of the visit and may further increase the intensity.

De-intensifying trial participation, together with a close and clear communication between the research team and trial participants may prevent early termination of trial participation [9].

Exchange of medical information between trial participants and the research team, using an mHDA prior to a trial visit, could alleviate the intensity burden. It may relief participants from the stress of recalling information on the spot and allow for more room to discuss relevant health issues. In addition, by exchanging information beforehand, the visit can be prepared by the research team, which guides the consultation and improves data consistency.

Previously, a systematic review on mobile phone apps for quality of life and well-being assessment in breast and prostate cancer patients reported that scientific literature, referring to mobile applications in trials, is scarce [10]. Nevertheless, via a prospective, randomised controlled trial on breast and prostate cancer, the use of an e-health platform has shown to improve clinical management, reduce costs and promote safe and participatory care [11].

To our knowledge, the use of an mHDA in the setting of CCTs with the purpose to improve data collection quality and efficiency has not yet been investigated.

This quantitative study aims to address the following:

The participant’s perspective: assessing the usability of an mHDA as a tool for providing medical information in advance to the research team.
The research team’s perspective: assessing the applicability of the provided information in the preparation of a participant’s visit and the usability of the shared information for an efficient and complete high quality data collection during the actual visit.

## Material and methods

This prospective observational study was approved by the Institutional Review Board of Maastro (W 18 12 00063) and was reported to the local ethics committee of Maastricht University Medical Centre. Based on the Dutch Central Committee on Research Involving Human Subjects (CCMO) regulations, the study was not subjected to the Medical Research Involving Human Subjects Act (WMO).

### Study population

Between April 2019 and February 2020, twenty trial participants in different radiation oncology trials were included in this study. The study population consisted of sixteen female and four male participants, with a mean age of 62 years (range 32–79 years). During the study period, a physician assistant and a radiation technologist with respectivelyseven and six years experience in clinical radiotherapy trial consultations were included for evaluation of the mHDA.

### Mobile health data capture application

The Improve app (Open HealthHub B.V., Utrecht, The Netherlands) used in this study, is an end-to-end encrypted mHDA, securing a direct and protected data transfer between participant and research team via an SSL connection [12]. In the Netherlands, the application is hosted by a NEN 7510 and ISO 27001 certified data centre. The application is designed to comply with Good Clinical Practice [13]. It is provided with a monitoring system to track returned questionnaires from individual participants.

The questionnaire used in the application was self-compiled, specific for this study. This generic list had to be applicable to participants from different clinical radiotherapy trials, questioning participants on general complaints, possibly related to their trial treatment. The structured list consisted of questions about most common health issues of all organ systems, current use of medication and a free text space (Appendix Fig. A and supplementary table A). Any new events were graded according to the Common Terminology Criteria for Adverse Events (CTCAE).

### Study procedure

With the study information, separate instructions for installing the application on iOS and Android platforms on participants’ smartphones were provided.

After the Informed Consent procedure, participants were included through the research teams’ interface of the mHDA (Fig. A.4). Participants were provided with a unique login code to enter the application, enabling to use the online questionnaire immediately after inclusion. A reminder to complete the questionnaire was sent two weeks before the appointment. It could be completed at any favourable moment and only after full completion, data were sent to the research team. Participants used the mHDA for one single appointment. During the participants’ actual visits, indicated health issues were addressed and if applicable, a physical examination was performed to investigate the complaints. If necessary, additional investigations were initiated.

### Evaluation

Participants received a Dutch printed version of the Systems Usability Scale (SUS) consisting of ten questions provided as a five-point Likert scale, to rate the usability and technical complexity of the application (Supplementary table B). The SUS is a quantitative questionnaire and a widely applied instrument for measuring usability of eHealth applications, using positive and negative questions to minimise bias [14], [15]. For participants who missed one or more scores on the SUS questionnaire, the ‘neutral’ option of 3 points was chosen. The SUS was modified by replacing the word ‘system’ in the original SUS by the word ‘app’.

Additionally, participants were provided with five questions on a five-point Likert scale to rate the efficiency and effectiveness of their visit (Supplementary table C).

Members of the research team reflected on the applicability of the application in the preparation of and its efficiency during appointments. The interview consisted of five general questions about former visits, three questions about the functionality of the application, six questions about toxicity related to the trial treatment, six questions about any new health issues not related to the trial, and three closing questions about the visit (Supplementary table D).

### Statistical analysis

The mean +/− standard deviation (SD) of the SUS is reported. To calculate individual SUS scores, uneven numbered questions are scored as x-1 and even numbered questions as 5-x, where x is the individual score on the five-point Likert scale. The total SUS score is a number between 0 and 100 and is calculated by adding all individual scores and multiplying them by 2.5 [16], [17].

A publication by Bangor et al. [18] describes the SUS scoring related to the performance of a system. They conclude that a system, scoring above 85 can be qualified as excellent.

To assess the applicability and efficiency for the research team, descriptive statistics are used.

## Results and discussion

### Study population, feasibility and performance

From the 20 participants, 18 used the mobile application, answering the online questionnaire completely and returning it to the research team. One participant did not receive a login code, another participant postponed the visit, exceeding the inclusion period. These two participants did not return the SUS. Another two participants used the mobile application after the visit and did not return the SUS. One participant used the application properly but did not return the SUS. This participant was included in the research teams’ evaluation, resulting in 16 evaluations on efficiency of participants’ visits. A total of 15 returned SUS forms were used in the analysis of this study.

### Systems usability scale and participants’ perspective

**Fig. B.1:**
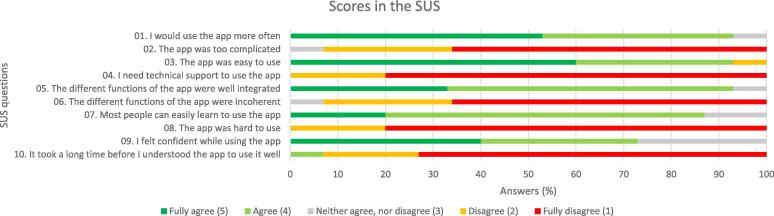
Participants’ ratings on the ten items from the Systems Usability Scale (SUS).

**Fig. B.2:**
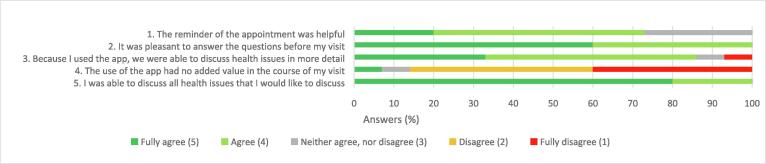
Participants’ ratings on the efficiency and effectiveness of their visit.

The results of the calculated SUS scores are displayed in Table A. The mean SUS score was 86.8 +/- 11.5, qualifying the mHDA as excellent. The scores from the five-point Likert scale on the ten questions from the SUS are displayed in Fig. B.1. The results of the additional five questions about the participants’ perceptions on efficiency and effectiveness of the visit are displayed in Fig. B.2.

Participants stated that it was useful to answer and return questions about their actual health status prior to their appointment. By using the application, participants stated that they had the opportunity to discuss all health issues in more detail, leading to a complete, more efficient and effective visit within the available amount of time. This observation is in line with literature, where utilisation of mobile applications is generally accepted. However, educating users adequately is critical for sustained use [19], [20], [21]. Based on the current study, the use of the mHDA was effective in de-intensifying participants’ visits.

The SUS used in this study is an easily applicable instrument for calculating the usability of eHealth applications. Other instruments are the Mobile App Rating Scale (MARS) [22], the Telehealth Usability Questionnaire (TUQ) [23] and the mHealth App Usability Questionnaire (MAUQ) [24]. The MARS could be a good alternative, but may be too extensive for evaluation of the mHDA in our study. The TUQ and the MAUQ consist, different from the SUS, of solely positive questions, increasing the risk of bias.

### Functionality of the application and effectiveness for the research team.

The mHDA was stable without loss of data during transfer, leading to a complete collection of answers.

Sixteen out of twenty participants returned their answers in time, enabling the researchers to prepare the participant’s visit. The results of the reported effectiveness is displayed in Fig. C. Average time of the visits was 31 min (range 15–45 min).

For the research team, the core functionality of the application was met. By receiving the participants’ medical information in advance, they were able to 1) address indicated health issues as possibly related to the treatment in the clinical trial, or as other new health issues not necessarily related and 2) discuss these issues with the participants, 3) acknowledge these issues by performing a physical examination, 4) present a differential diagnosis and 5) agree upon a policy for further treatment or investigation.

Literature on efficiency of mobile applications in the communication with patients varies among publications, most regarded as positive [25], [26], but some as less efficient [27].

This study has shown that the use of an mHDA can be useful and time-efficient; the research team was able to achieve a complete data assessment for the purpose of the clinical trial. Hence, the use of mHDA’s in a clinical trial setting may lead to a higher quality in the data assessment compared to the conventional way of data collection without the availability of medical information in advance.

Another way of digitally exchanging medical information can be performed by using ePRO’s. However, this way of data exchange encompasses an easily accessible system for following up quality of life issues. In clinical trials, toxicities may not be standard due to the alternative treatment, making a more robust and more flexible digital platform, providing bidirectional communication, recommendable to collect an extensive set of data. An mHDA may provide this solution. [28].

This study has some limitations; 1) the study was conducted with a limited number of participants. A higher inclusion may lead to more robust data. Nevertheless, the results in this pilot population were shown to be consistent in favour of the mobile application. Furthermore, 2) a control group was not included, making it difficult to draw conclusions about an improvement in the quality of the data collection. However, the application itself was given an excellent SUS score, indicating that the application was easy to use. Furthermore, the availability of the application was highly appreciated by the participants regarding the efficiency and effectiveness in the preparation of their visit. Also, for the research team the application was appreciated as an efficient tool in the way that the participants’ medical information was provided in advance, giving the research team the opportunity to prepare the visit and focus on for participants relevant topics.

An mHDA can improve the efficiency and effectiveness of participants’ follow-up visits in CCTs and hence improve the quality of the data collection in these trials. Further research in larger groups of participants is needed to answer this question.

## Declaration of Competing Interest

The authors declare that they have no known competing financial interests or personal relationships that could have appeared to influence the work reported in this paper.

## Figures and Tables

**Fig. A:**
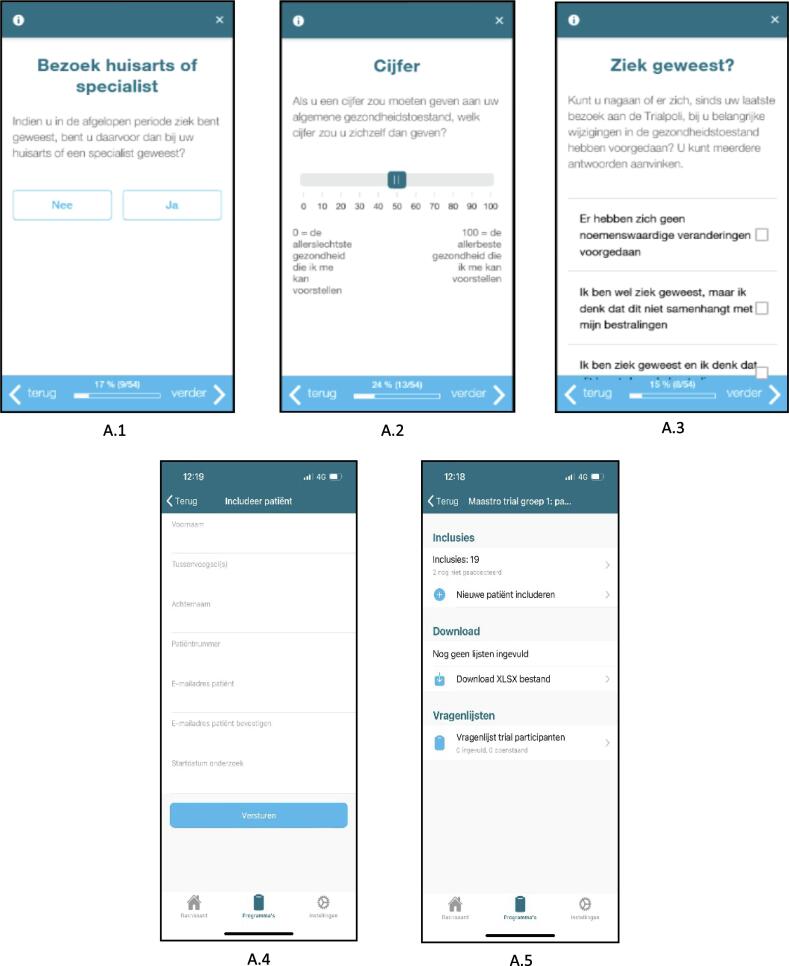
A.1 to A.3 screenshots of participants’ interface and A.4 and A.5 of research teams’ interface of the mobile data-capture application. Translation of the Dutch text on the next page.

**Table A:** Systems Usability Scale (SUS) scores per individual, total SUS score per individual and calculated mean SUS score.

Subject nr.	1	2	3	4	5	6	7	8	9	10	11	12	13	14	15	Mean SUS score (+/- SD)
1. I would use the app more often	3	4	4	3	4	3	3	4	3	4	4	2	3	4	4	
2.The app was too complicated	4	4	3	3	4	3	4	4	4	4	4	2	4	4	3	
3.The app was easy to use	4	4	4	4	4	3	4	4	3	4	4	1	3	3	3	
4. I need technical support to use the app	4	4	4	3	4	3	4	4	4	4	4	3	4	4	4	
5. The different functions of the app were well integrated	3	4	3	4	4	3	3	3	4	3	4	2	3	3	3	
6. The different functions of the app were incoherent	3	4	3	4	4	3	4	4	4	3	4	2	4	4	4	
7. Most people can easily learn to use the app	3	4	3	3	3	3	2	3	4	3	3	2	3	4	3	
8. The app was hard to use	4	4	4	4	4	3	4	4	4	4	4	3	4	4	3	
9. I felt confident while using the app	4	4	3	3	4	3	3	4	4	2	2	2	2	3	4	
10. It took a long time before I understood the app to use it well	4	4	4	4	4	3	4	4	4	4	4	1	4	3	3	
**Total SUS score per individual**	90	100	87.5	87.5	97.5	75	87.5	95	95	87.5	92.5	50	85	90	82	**86.8 (+/- 11.5)**

**Fig. C:**
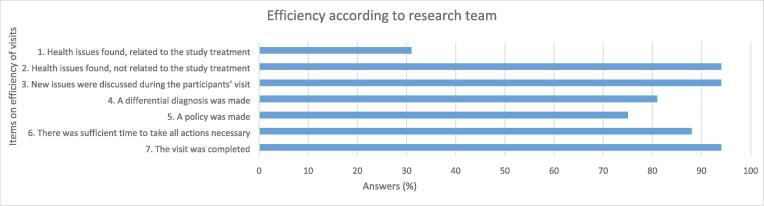
Visualisation of efficiency of participants’ visits according to researchers’ opinion.

## Appendix A

See Fig. A.

Translation of screenshots displayed in figures A.1 to A.5:

A.1: **Visit to a family physician or medical specialist**; in case you have been ill in the past period, did you visit your family physician or a medical specialist? **NO/YES**

A.2: **Score**; if you were asked to score your general health, which score would you give?

0 = worst imaginable health, 100 = best imaginable health

A.3: **Have you been ill**; can you recall if there have been any major changes to your health since your last visit to the trial outpatient clinic? You can check more than one box.

• There have been no important changes
• I have been ill, but I think there is no relation to my radiotherapy treatment
• I have been ill and I think it was related to my radiotherapy treatment…

The complete questionnaire is added as supplementary table A.

A.4: **Include patient;**

First name

Insertion

Last name

Patient number

E-mail address patient

Confirm e-mail address patient

Start date trial

Blue button: Send

A.5: **Maastro Trial group 1;**

**Inclusions**

Inclusions: 19

2 not yet excepted

+ Include new patient

**Download**

No lists completed

Download XLXS file

**Questionnaires**

Questionnaire trial participants

0 completed – 0 waiting

See Table A and Fig. B.1, Fig. B.2, Fig. C.
