# Supplementary material for: Performance and usability evaluation of a mobile health data capture application in clinical cancer trials follow-up
Source: Tech Innov Patient Support Radiat Oncol. 2022 Nov 1;24:107–12. doi: 10.1016/j.tipsro.2022.10.005 (PMC9641052; doi:10.1016/j.tipsro.2022.10.005)
Supplement: Supplementary data 1 [file mmc1.docx]

Supplementary material to the short paper entitled:

**Performance and usability evaluation of a mobile health data capture application in clinical cancer trials follow-up**

**Supplementary table A:**

Participants’ questionnaire in the mobile data-capture application

| Question | Type |
| --- | --- |
| In this questionnaire we like to be informed about changes is your general health compared to your last visit to our outpatient clinic. Take your time to recall if there have been any changes regarding your health in the past period. Almost all questions can be answered by the YES or NO button on your screen.  During your visit to the trial outpatient clinic we can discuss these new health issues in more detail. The purpose of this app is that we have more time to discuss these issues during your visit. Good luck! | General information |
| First, we would like to know if there have been any changes in your medication. You can use your medication passport from your pharmacist or the packages of your medication. | General information |
| Has there been any change in your medication since your last visit? | Yes/No |
| Can you provide a list of changes in your medication? | Free text field |
| Now, we have some questions about your general health. Some complaints can be related to your radiation treatment. | General information |
| Can you recall if there have been major changes to your health since your last visit to our outpatient clinic? There are more answers possible. | More answers possible :   - There have been no noteworthy changes to my health - I have been ill but I think this had nothing to do with my radiotherapy treatment - I have been ill and I think this was due to my radiotherapy treatment - Since my radiotherapy treatment I’m tired and this does not improve - After my radiotherapy treatment I was tired, but I have recovered from that |
| If you have been ill in de past period, did you visit your family practitioner or a doctor in your hospital? | Yes/No |
| Do you smoke? | Yes/No |
| Do you drink alcoholic beverages? | Yes/No |
| Are you on drugs | Yes/No |
| If you were asked to give a rating to your health, which number would you give? | Rating on a scale from 0 to 100 in which 0 equals the worst imaginable health and 100 equals the best imaginable health |
| Now we have some more specific questions about your general health. Please note that all questions reflect the period since your last visit to our outpatient clinic. All questions can be answered with a yes or no | General information |
| Did you encounter any new allergies? | Yes/No |
| Are there any new lesions on your skin or is there any rash | Yes/No |
| Does your skin itch? | Yes/No |
| Did you notice any hair loss | Yes/No |
| Did you experience any tingling in your fingers, hands, soles of the feet, you face or on your head? | Yes/No |
| Do you experience any numb feeling | Yes/No |
| Do you experience loss of muscle power or weakness? | Yes/No |
| Do you experience any headache? | Yes/No |
| Do you experience any dizziness? | Yes/No |
| Do you experience any palpitations? | Yes/No |
| Do you experience any chest pain? | Yes/No |
| Did you notice a swollen, red leg in the past period? | Yes/no |
| Do you experience any pain in your leg while walking | Yes/No |
| Did you notice any shortness of breath in the past period? | Yes/No |
| Do you sleep well? | Yes/No |
| Do you sleep on one pillow? | Yes/No |
| Do you experience swollen ankles? | Yes/No |
| Do you have to get out of bed at night to urinate | Yes/No |
| Do you need to cough more frequently than usual? | Yes/No |
| Do you cough up mucus? | Yes/No |
| Has the sound of your coughing changed? | Yes/No |
| Have you experienced a pneumonia in the past period? | Yes/No |
| Do you have a normal appetite? | Yes/No |
| Are you able to swallow in a normal way? | Yes/No |
| Have you been nauseous over the last period and did you need to vomit | Yes/No |
| Do you experience any small ulcers in your mouth? | Yes/No |
| Did you lose or gain any weight over the past period? | - I gained weight - I lost weight - Neither |
| Did you stool change over the past period? | Yes/No |
| Did you notice any blood in your stool? | Yes/No |
| Did you notice black colored stool | Yes/No |
| Are you able to urinate normally | Yes/No |
| Did you notice any blood in your urine? | Yes/No |
| Do you experience any urinary incontinence? | Yes/No |
| Do you experience any complaints of your joints | Yes/No |
| Are you able to move easily after you have rested? | Yes/No |
| Do you experience unexplainable pain in muscles or tendons | Yes/No |
| If there are any other health complaints would please register them in this field? | Free text |
| Have there been any major changes or dramatic events in your live over the past period | Yes/No |
| If you may have any questions or remarks which have not been discussed in this questionnaire, please leave them here so we can discuss them during your visit | Free text |
| This was the last question. You can send them by clicking on the ‘send’ button |  |

**Supplementary table B:**

Systems Usability Scale (SUS)

|  | **Fully disagree** | **Disagree** | **Neither agree, nor disagree** | **Agree** | **Fully agree** |
| --- | --- | --- | --- | --- | --- |
| I think I would use the app more often | 1 | 2 | 3 | 4 | 5 |
| I think the app was too complicated | 1 | 2 | 3 | 4 | 5 |
| I think the app was easy to use | 1 | 2 | 3 | 4 | 5 |
| I think I need technical support to use the app | 1 | 2 | 3 | 4 | 5 |
| I think the different functions of the app were well integrated | 1 | 2 | 3 | 4 | 5 |
| I think the different functions of the app were incoherent | 1 | 2 | 3 | 4 | 5 |
| I think most people can easily learn to use the app | 1 | 2 | 3 | 4 | 5 |
| I think the app was hard to use | 1 | 2 | 3 | 4 | 5 |
| I felt confident while using the app | 1 | 2 | 3 | 4 | 5 |
| It took a long time before I understood the app to use it well | 1 | 2 | 3 | 4 | 5 |

**Supplementary table C:**

Additional questions for evaluating the efficiency and effectivity of participants’ visit

|  | **Fully disagree** | **Disagree** | **Neither agree, nor disagree** | **Agree** | **Fully agree** |
| --- | --- | --- | --- | --- | --- |
| I think the reminder of the appointment was helpful | 1 | 2 | 3 | 4 | 5 |
| I think it was pleasant to answer the questions before my visit | 1 | 2 | 3 | 4 | 5 |
| Because I used the app, we were able to discuss health issues in more detail | 1 | 2 | 3 | 4 | 5 |
| The use of the app had no added value in the course of my visit | 1 | 2 | 3 | 4 | 5 |
| I was able to discuss all health issues that I would like to discuss | 1 | 2 | 3 | 4 | 5 |

**Supplementary table D:**

Research teams’ questions for evaluating participants’ use of the application and the efficiency of the application in the preparation of the appointment

| **Question** | **Result** |
| --- | --- |
| ***General*** | |
| In which trial did the participant take part? | Name trial |
| What was the diagnosis of the participant in the trial: | Participants’ diagnosis |
| In which trial arm (if applicable) was the participant allocated: | Allocation |
| Has any early or late toxicity been measured in former visits?  If applicable, what were these toxicities? | Yes/no  Addition |
| Did the participant experience former health issues, not related to the illness for which the participant was treated within the trial?  If applicable, where did these health issues consist of | Yes/no  Addition |
| ***Questions about the use of the application*** | |
| How many questions did the participant answer by means of the application? | ……../44 = ………% |
| Did the participant return the answers to the research team?  If not, what was the reason?  Give an explanation | Yes/no  Technical problem/forgotten/other  Addition |
| Did you receive the answers on your smartphone?  If not, what was the reason?  Give an explanation | Yes/no  Technical problem/forgotten/other  Addition |
| ***Toxicity related to the trial*** | |
| Was any new toxicity, related to the treatment within the trial,  discussed | Yes/no |
| Did the participant indicate this in the app | Yes/no |
| If applicable, did you question the participant about the new toxicity? | Yes/no |
| If applicable, did you perform a physical examination (PE)?  If not, why not?  If yes, what PE was performed? | Yes/no  Addition  Description of PE |
| If applicable, was a differential diagnosis made on basis of anamnesis and PE? | Yes/no |
| If applicable, was a policy made on basis of anamnesis and PE? | Yes/no |
| ***New health issues, not formerly mentioned*** | |
| Were any new health issues discussed? | Yes/no |
| Did the participant indicate this in the app? | Yes/no |
| If applicable, did you question the participant about the new health issues? | Yes/no |
| If applicable, did you perform a physical examination (PE)?  If not, why not?  If yes, what PE was performed? | Yes/no  Addition  Description of PE |
| If applicable, was a differential diagnosis made on basis of anamnesis and PE? | Yes/no |
| If applicable, was a policy made on basis of anamnesis and PE? | Yes/no |
| ***Closing questions*** | |
| How long did the visit take? | Indicate minutes |
| Did you have enough time for a complete visit?  If not, why not? | Yes/no  Addition |
| To your opinion, did you perform everything that was necessary? | Yes/no |

**Supplementary table E:**

Participants of the mobile application study and the radiotherapy trials they are enrolled in

| **Trial** | **Title** | **Clinicaltrials.gov** | **Nr. Of participants** |
| --- | --- | --- | --- |
| EORTC 26053_22054 CATNON Intergroup Trial | Phase III trial on Concurrent and Adjuvant Temozolomide chemotherapy in non-1p/19q deleted anaplastic glioma | NCT00626990 | 3 |
| IRMA trial, | Breast cancer with low risk of local recurrence: partial and accelerated radiation with three-dimensional conformal radiotherapy (3DCRT) versus standard radiotherapy after conserving surgery; phase III study | NCT01803958 | 11 |
| PROBACH Trial | Randomized Phase 3 Study On The Assessment Of Late Toxicity By Comparing IMRT High Dose External Beam Radiotherapy Only With External Beam Radiotherapy Combined With HDR Or PDR Brachytherapy In Patients With Intermediate/high Risk Prostate Cancer |  | 2 |
| ART DECO Trial | A Randomized Trial of Dose Escalation in definitive Chemoradiotherapy for patients with esophageal cancer |  | 1 |
| EORTC 22042_26042 MENINGIOMA trial | Adjuvant postoperative high-dose radiotherapy for atypical and malignant meningioma: a Phase II and observation study | NCT00626730 | 1 |
| BOOG 2004-01 CTKO 2003-13 YOUNG BOOST TRIAL | Radiation dose intensity study in breast cancer in young women; a randomized phase III trial of additional dose to the tumor bed | NCT00212121 | 2 |
